# Mobile EEG examination of putative biomarkers of mental health in individuals that use cannabis

**DOI:** 10.1038/s44277-025-00039-8

**Published:** 2025-07-03

**Authors:** Conor H. Murray, Kaihan Danesh, Ziva D. Cooper

**Affiliations:** 1 UCLA Center for Cannabis and Cannabinoids, Jane and Terry Semel Institute for Neuroscience and Human Behavior, Department of Psychiatry and Biobehavioral Sciences, David Geffen School of Medicine, University of California, Los Angeles, CA USA; 2 Department of Anesthesiology and Perioperative Medicine, David Geffen School of Medicine, University of California, Los Angeles, CA USA

**Keywords:** Predictive markers, Cognitive neuroscience

## Abstract

Healthy individuals that use cannabis are at greater risk of developing mental health conditions than those that do not use cannabis. Here, using mobile electroencephalography (EEG) in controlled laboratory settings, we examined two putative biomarkers of mental health across two studies of people who use cannabis (*N* = 100, 50% male; *N* = 40, 60% male). We examined associations to cannabis use and mood and assessed the influence of sex and age on the outcomes. Specifically, in the first study, we examined prefrontal broadband power, previously found to be related to healthy neurocognitive development, in relation cannabis use. We also examined left prefrontal alpha power, previously found to be related to anxiety and depression, in relation to Beck Depression Inventory (BDI) and Beck Anxiety Inventory (BAI) scores. In the second study, we examined only left prefrontal alpha power during the cold pressor test (CPT), which elicits a stress response. We found that in the first study, young males (ages 21–23) showed the greatest association between prefrontal broadband power and cannabis use (R = 0.50; *p* = 0.007), while females showed associations between left prefrontal alpha power and BAI scores (R = 0.61, *p* = 0.013). In the second study, the CPT increased anxiousness (*p* < 0.001) but did not affect left prefrontal alpha power. Together, our findings help to characterize these putative biomarkers in individuals that use cannabis, while informing the utility of mobile EEG devices for tracking markers of mental health and wellness outside of laboratory settings.

## Lay summary

This manuscript includes data from individuals who use cannabis who visited the Center for Cannabis and Cannabinoids at UCLA. Researchers recorded 5 minutes of brain activity from 100 individuals during eyes closed rest using a “brain sensing headband,” a mobile electroencephalography (EEG) device. Researchers examined EEG markers of cognitive and emotional wellbeing, finding that in males, self-reported cannabis use was associated with reduced cognitive wellbeing, as indexed by the EEG device. In females, self-reported anxiety was associated with reduced emotional wellbeing, as indexed by the EEG device. In 40 additional individuals, a stress test was used to induce anxiety acutely, however, this did not affect the EEG measure of emotional wellbeing, indicating that the EEG measure may relate to individual differences in emotional wellbeing more than state-dependent changes in emotional wellbeing. The findings inform the utility of EEG and mobile EEG in tracking markers of brain health.

## Introduction

Recent advancements in digital technologies have resulted in a growing adoption of electroencephalography (EEG) and other devices for use by the public as part of broader trends in biofeedback and wellness. EEG is the principal diagnostic tool for epilepsy, identifying abnormal electrical activity characteristic of seizure disorders. EEG is also used in clinical settings to monitor brain activity related to depth of unconsciousness, including coma or anesthesia during surgery. The use of EEG to identify biomarkers of mental health is an active area of research [1]. For instance, EEG has shown potential to aid in the diagnosis of developmental and cognitive disorders including autism, ADHD, and schizophrenia, as well as depression and anxiety. In addition, the use of machine learning on EEG data was recently able to identify or classify individuals that use heroin with 97% accuracy [2].

Building on this momentum, researchers have begun exploring the utility of EEG in populations at elevated risk for mental health challenges, including individuals who use cannabis. Cannabis use in otherwise healthy individuals has been linked to a range of cognitive and emotional health outcomes [3], yet relatively few studies have used EEG to investigate brain-based biomarkers in this population. Our work, and the work of others, have uncovered two putative EEG biomarkers related to cognitive and emotional health that may be associated with cannabis use. First, our work involving a putative marker related to cognitive health, examined resting state EEG in adolescent (ages 18–20) and adult (ages 20–30) individuals with less than 10 total lifetime exposures of cannabis use [4]. We found that the adult group had significantly less oscillatory power across frequency bands (broadband; delta, theta, alpha, beta, and gamma) under prefrontal electrodes of a 128 electrode EEG system, indicating greater asynchronous brain activity in the prefrontal cortex at rest after healthy brain development. Generally, the power of neural oscillations increases when neural circuits are unperturbed, synchronizing during sleep (delta, theta), rest (alpha), or focused attention (beta, gamma), whereas the introduction of exogenous stimuli, or otherwise endogenous brain activity, desynchronizes these waveforms to reduce their amplitude and power [5]. Our findings, showing that resting state prefrontal broadband power desynchronizes from adolescence to adulthood, is supported by earlier findings from mobile EEG devices, which are lightweight, wireless EEG systems that enable rapid and scalable data collection. Specifically, using Muse mobile EEG devices, researchers observed a neurodevelopmental trajectory of prefrontal broadband power indicating that asynchronous prefrontal brain activity peaks after brain maturation near middle age, before declining in late adulthood [6]. Together, prior work indicates that prefrontal broadband power is a putative marker of cognitive health related to neurodevelopment and aging. Second, involving a putative marker related to emotional health, prior work examining resting state EEG in individuals with depression and anxiety demonstrates possible asymmetries in frontal alpha power between hemispheres, though findings have been mixed [7, 8]. Some studies have found greater alpha power in left prefrontal cortex associated with depression and anxiety [7, 9]. Mechanistically, as alpha oscillations synchronize during periods of inactivity and rest [10–14], greater alpha power in the left cortex is thought to reflect impaired activation of top-down cognitive control over subcortical emotion-related responses, including outputs from the amygdala [9, 15, 16].

Here, we examined two putative biomarkers related to cognitive and emotional health, i.e., prefrontal broadband power and left prefrontal alpha power, respectively, in individuals that use cannabis, a population at risk of cognitive and emotional health conditions [3]. In the first study, we hypothesized that greater cannabis use would be associated with reduced prefrontal brain activity at rest, based in part, on preclinical findings of impaired maturation of the prefrontal cortex in rodents exposed to cannabinoids [17]. With respect to emotional health, we hypothesized that greater scores on the Beck Depression Inventory (BDI) and Beck Anxiety Inventory (BAI) would be associated with left prefrontal alpha power. Further, in a second study, we hypothesized that anxiety may be induced experimentally with the cold pressor test (CPT), an experimental test known to elicit a stress response, and that EEG recordings obtained during the CPT might show a modulation of left prefrontal alpha power. In all EEG analyses, we used Muse mobile EEG headbands because these devices (1) have been validated to detect the two putative biomarkers in prior studies [6, 18] (2) enable rapid data collection, which was required for obtaining our measures, as this occurred during participant screening visits prior to studies involving cannabis and cannabinoid administration, and (3) inform real-world applications for tracking brain health for individuals in naturalistic settings. Together, our objective was to determine whether putative biomarkers of cognitive and emotional health relate to measures of cannabis use and mood and whether these relationships occurred in a sex- and age-dependent manner.

## Materials and methods

### Participants

Participants were individuals between the ages of 21–55 (*N* = 100, Study 1; *N* = 40, Study 2) who visited the laboratory after responding to advertisements and passing a telephone screen of inclusion/exclusion criteria. Specifically, participants were included if they reported current cannabis use (within the last 30 days). Participants were excluded if cannabis use predominately reflected use of medical cannabis or were currently prescribed for pain relievers or other medications, including psychiatric medications, that may affect study outcomes. In addition, participants who reported the use of other drugs in the last 30 days, except for alcohol or tobacco/nicotine, were also excluded from participation. All participants visited the UCLA Center for Cannabis and Cannabinoids for a single visit involving an interview, surveys, mobile EEG, and CPT. In addition to providing measures for the current analysis, this laboratory visit also served as an in-person screening visit ahead of additional laboratory studies of cannabis and cannabinoid administration. Our research was undertaken with the understanding and written consent of each subject in accordance with the Declaration of Helsinki and all screening and study procedures were approved by the UCLA Institutional Review Board (IRB-19-0876, IRB-19-1519, IRB-21-1137, IRB-21-0208).

### Cannabis use measures

All participants were interviewed by research staff to assess prior substance use and mental health. Several measures relevant to cannabis use were collected as previously described [4], including self-reported total days of lifetime cannabis use, years since first use, and days since last cannabis use. To operationalize cannabis use within a single measure for our EEG analyses, for each participant, total lifetime cannabis use was divided by the years since the initiation of cannabis use, resulting in a number from 0–365 days, indicating each participant’s average yearly cannabis use.

### Anxiety and depression measures

The Beck Anxiety Inventory (BAI) and Beck Depression Inventory (BDI) are participant-rated assessments to obtain scores of anxiety and depressive symptoms over the past month and week, respectively. BAI scores indicate minimal (0–7), mild (8–15), moderate (16–25) and severe (26–63) anxiety symptoms (Beck et al. [19]), while BDI scores indicate minimal (0–13), mild (14–19), moderate (20–28) and severe (29–63) depressive symptoms (Richter et al. [20]).

### Other substance use measures

Our interview asked about current alcohol use and tobacco use, specifically the number of drinks of alcohol per week and number of cigarettes per day, which we incorporated in our analysis to address the specificity of findings on cannabis use as opposed to substance use broadly. Other measures specific to substance use included the Cannabis Use Disorder Identification Test - Revised (CUDIT-R; [21]) and Michigan Alcohol Screening Test, short form (MAST; [22]). CUDIT-R scores of 8 or more indicate hazardous use; while 12 or more indicates the need to assess for cannabis use disorder. A MAST score of 0–3 suggests lack of alcohol dependence.

### Other mental health measures

Additional measures included the Trauma Assessment for Adults (TAA; Resnick et al. [23]), and Yale University PRIME Screening Test (Yale; Miller et al. [24]). The TAA consists of 13 items (e.g., “Combat”) inquiring about the experience of potentially traumatic events, without a threshold score. Yale scores of 1–5 indicate non-psychotic severity, with a score of 6 indicating “severe and psychotic.”.

### Cold pressor test and state anxiety

In the second study (*N* = 40), participants completed the cold pressor test (CPT), an experimental test that elicits a stress response (Siegel et al. [25]). Specifically, participants were instructed to rest their hand first in a vat of warm water (37 °C) for 3 min. Then, participants moved the same hand to the cold water (4 °C) and were instructed to inform research staff when the experience of the cold water became unbearable and the hand needed to be removed. The CPT provided an opportunity to examine experimentally-induced anxiety during our investigation of EEG markers of emotional health, specifically feelings of anxiousness. The state of induced anxiety associated with both the cold and warm water conditions was operationalized with the state portion of the State-Trait Anxiety Inventory (STAI; Speilberger [26]) immediately following the CPT, with “moderate anxiety” scored in the 38–44 range.

### Mobile EEG recordings and analyses

All EEG recordings were obtained in a controlled environment, specifically a small single participant session room in the laboratory. Muse S headbands (Interaxon, Ontario, CA) were used to record electrical activity under anterior-frontal (AF) 7 and AF8 sensors, corresponding to electrical activity under the left and right hemispheres of the prefrontal cortex. Temporoparietal (TP) 9 and TP10 sensors are also included in the headband, which were not used in our analyses, but aided in quality assurance during device testing. The reference electrode in Muse is the Fpz electrode. The Muse S EEG recordings were obtained via mobile application through the Mind Monitor app (previously known as the Muse Monitor app). The Mind Monitor app (mind-monitor.com) was developed for academic neuroscience research with Muse devices, measuring the logarithm (log) of the power spectral density in units of microvolts squared per Hz (μV^2^/Hz). Each EEG file was saved as a .CSV file and opened into MATLAB and EEGLAB via the Muse Monitor plugin extension. Mind Monitor utilizes an EEGLAB plugin with automated artifact rejection techniques, including filtering and rejecting data segments using Artifact Subspace Reconstruction (ASR). The EEG recordings were also visually inspected in the EEGLAB graphical user interface for artifacts as previously described [27]. No independent components analysis (ICA) was performed here as only four electrodes are available through the Muse S system, limiting the utility of ICA. We used the same custom script in previous studies [4, 27, 28] to examine the log of the EEG spectral power under each electrode across frequency bands over the entire recording period (delta, 1–4 Hz; theta, 4–8 Hz; alpha 8–13 Hz; beta 13–30 Hz; gamma 30–80 Hz).

In the first study (*N* = 100), mobile EEG was used to record 5 min of eyes closed resting state activity with participants alone in the session room. This activity was examined in relation to cannabis use, BDI and BAI. Specifically, a putative marker of cognitive health, the asynchronous activity across all frequency bands (reduced delta, theta, alpha, beta, and gamma power) averaged under the AF7 and AF8 sensors, was examined against cannabis use. A putative marker of emotional health, alpha power (8–13 Hz) under the AF7 sensor, was examined against BDI and BAI scores [29]. In the second study (*N* = 40), mobile EEG was used to record 3 min of eyes closed brain activity in the presence of a researcher during the warm water phase of the CPT and then again during the cold water phase of the CPT until the point that pain tolerance was reached.

In the second study (*N* = 40), the putative marker of emotional health was examined during both the warm- and cold-water conditions against STAI scores associated with the warm and cold water.

### Statistical analyses

All statistical analyses were conducted with SPSS (version 25; SPSS Inc, Chicago, IL). Pearson correlations were used to relate EEG measures to cannabis use, BDI, BAI, or STAI measures. The Pearson correlation coefficient (*r*) measures the strength and directionality of the linear relationship, where *r* = 1.0 is a perfect positive linear relationship, *r* > 0.7 is strong, *r* > 0.5 is moderate, and *r* > 0.3 is a weak correlation [30]. To account for potential confounds on the relationships, we conducted moderation analyses in SPSS (PROCESS version 4.2) specifically to test for interactions with alcohol use (drinks per week), tobacco use (cigarettes per day), and recent cannabis use (days since last cannabis use) on the EEG measures, left frontal alpha power and broadband frontal power. In addition, we accounted for broadband frontal power in our examinations of left frontal alpha power. Two-tailed t-tests were used to assess for differences in EEG measures between groups that were median split on the variables of cannabis use, BDI, and BAI. Post-hoc correlations assessed differences in EEG measures as a function of age and sex to address potential age- and sex-differences related to risks of cannabis use [4, 31]. To assess age differences, within SPSS, we binned participants into three age groups using two cut points containing equal percentiles based on scan cases, which is designed to divide the data into approximately equal-sized groups. The binning created three age groups (ages 21–23, 24–29, and 30–55 years; Ns = 33, 30, 37, respectively). Paired t-tests, two-tailed, were used to assess differences in EEG and STAI measures between the periods of warm- and cold-water hand immersion.

## Results

### Validation

We first validated the sensitivity of our Muse device’s sensors during a 5 min. eyes closed resting state on a single volunteer. The result of this quality assurance test (Supplementary Fig. 1) showed prominent alpha peaks in the 8–13 Hz range under the temporoparietal sensors (TP9, TP10), but not the anterior-frontal sensors (AF7, AF8), which we anticipated due to prominent alpha activity in close proximity to the occipital cortex during eyes closed conditions. These data provided evidence that our particular Muse device should reliably detect the two putative biomarkers we were interested in examining in individuals that use cannabis.

### Study 1

**Fig. 1:**
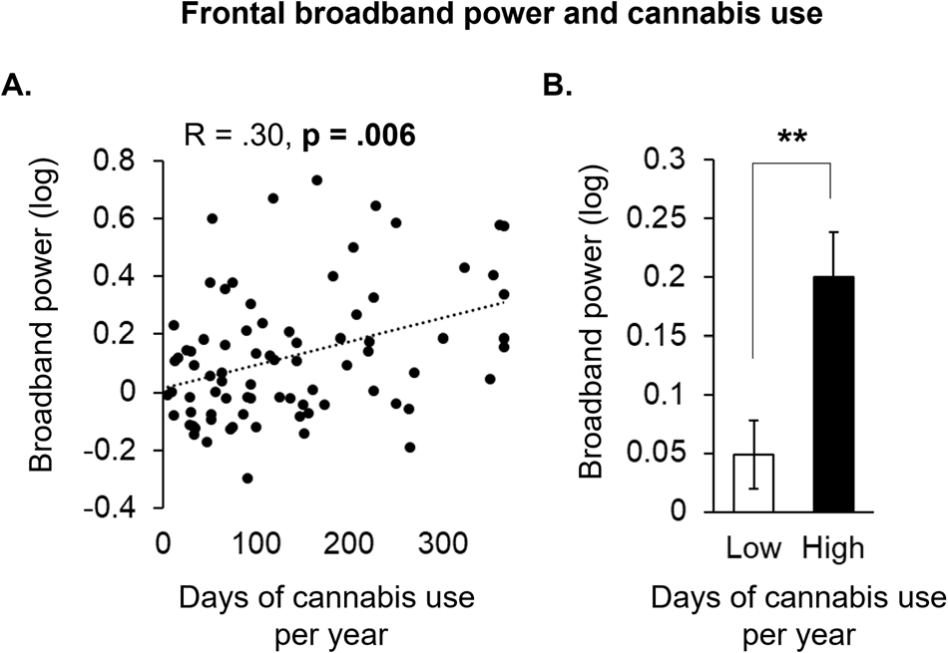
Frontal broadband power increases as a function of cannabis use. Scatter plot (**A**) showing delta, theta, alpha, beta, and gamma power (broadband power) averaged under the AF7 and AF8 sensors against the average days of cannabis use per year for each participant. Median split bar graph (**B**) of the participants in A along the continuum of days of cannabis use per year comparing individuals with lower (Low) relative to higher (High) cannabis use (*n* = 100; **p < 0.01).

In the first study (Demographics shown in Table 1), we found that the average number of days of cannabis use per year was associated with greater synchrony across delta, theta, alpha, beta, and gamma frequency bands (i.e., more broadband power) averaged under AF7 and AF8 sensors (*r* = 0.30; *p* = 0.006; Fig. 1A), indicating that cannabis use is associated with less asynchronous brain activity in the prefrontal cortex. A median split on the average number of days of cannabis use per year further indicated a significant difference in prefrontal activity between individuals with “Low” (mean: 53.31; SD: 28.62) relative to “High” (mean: 219.52; SD: 83.94) days of cannabis use (*p* = 0.009; Fig. 1B). The demographic characteristics of the “Low” and “High” groups are shown in Supplementary Table 1. To check for a potential influence of recent cannabis use, we conducted a moderation analysis incorporating the days since last cannabis use as an interaction term, finding no interaction to recent use (*p* = 0.208).

**Table 1:** Demographic characteristics of participants in Study 1, which used mobile EEG during 5 min of eyes closed resting state.

Category	*N* or Mean ± SD (Range)
Subjects (Male/Female)	100 (50/50)
Age, Years	29 ± 8 (21–55)
Body Mass Index, kg/m^2^	24 ± 4 (16–33)
Cannabis use	
Age first use, Years	17 ± 3 (7–27)
Years of use, Years	13 ± 9 (2–43)
Total lifetime use, Days	1974 ± 2944 (20–20,000)
Average days of use per year, Days	136 ± 104 (5–365)
CUDIT-R score	11 ± 5 (1–24)
Alcohol use (% weekly)	63%
MAST score (of weekly)	0.8 ± 1.3 (0–8)
Drinks per week (of weekly)	5.5 ± 5 (0–21)
Tobacco use (% daily)	20%
Cigarettes/day (of daily)	5 ± 2.6 (2–7)
Psychiatric self-assessment	
Beck Depression Inventory	4.7 ± 5.6 (0–34)
Beck Anxiety Inventory	6.2 ± 7.8 (0–42)
Trauma score	1.3 ± 1.3 (0–5)
Yale psychosis score	22 ± 11 (11–64)

*CUDIT-R* cannabis use disorder identification test – Revised, *MAST* michigan alcohol screening test.

We then addressed the specificity for the relationship between increased prefrontal broadband power to cannabis use by examining relationships to the number of alcoholic drinks per week, or cigarettes per day, in the subset of participants who reported current alcohol and cigarette use, finding no association with broadband power averaged under AF7 and AF8 sensors to alcohol use (*n* = 63; *r* = 0.16; *p* = 0.20) or tobacco use (*n* = 20; *r* = 0.06; *p* = 0.80). Furthermore, we conducted moderation analyses to assess the impact of alcohol drinks per week and cigarettes per day on the relationship to prefrontal broadband power, finding no significant interactions with alcohol (*p* = 0.213) or tobacco (*p* = 0.844).

**Fig. 2:**
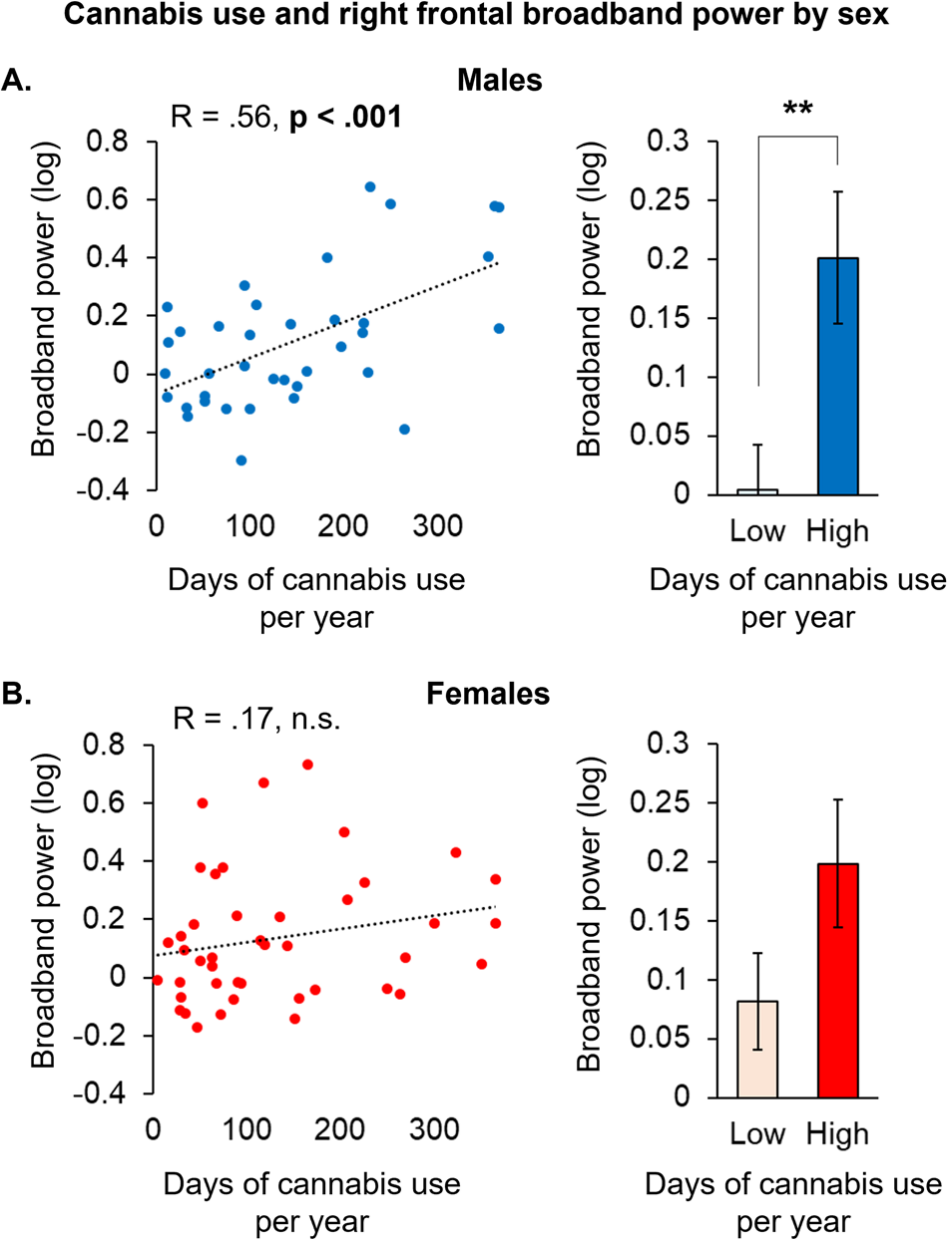
Increased frontal broadband power associated with cannabis use is sex-dependent. Scatter plots and median split bar graphs of data from the male (**A**) and female (**B**) participants showing associations between broadband power under the AF8 sensor relative to the average days of cannabis use per year (*n* = 100; **p < 0.01).

We next sought to explore whether the correlation between cannabis use and prefrontal EEG broadband power (less asynchronous activity) depended on either the left or right hemisphere. We found that the correlation to cannabis use was significant under the right, AF8 sensor (*r* = 0.37; *p* < 0.001), but not under the left AF7. Post-hoc analyses examining relationships to cannabis use under the AF8 sensor as a function of sex revealed a moderate correlation in males (*r* = 0.56; *p* < 0.001) (Fig. 2A), which was absent in females (Fig. 2B). Within males, the association between cannabis use and prefrontal EEG broadband power was most robust in young age, strengthening from a weak and non-significant correlation (*r* = 0.42; *p* = 0.096), to a moderate, but non-significant correlation (*r* = 0.56; *p* = 0.111), to both a strong and significant correlation (*r* = 0.70; *p* = 0.012) across the 30–55, 24–29, and 21–23 age groups, respectively (Supplementary Fig. 2A). Notably, there were no differences in reported cannabis use between the male and female groups, or between the youngest and oldest age groups (Supplementary Fig. 3).

**Fig. 3:**
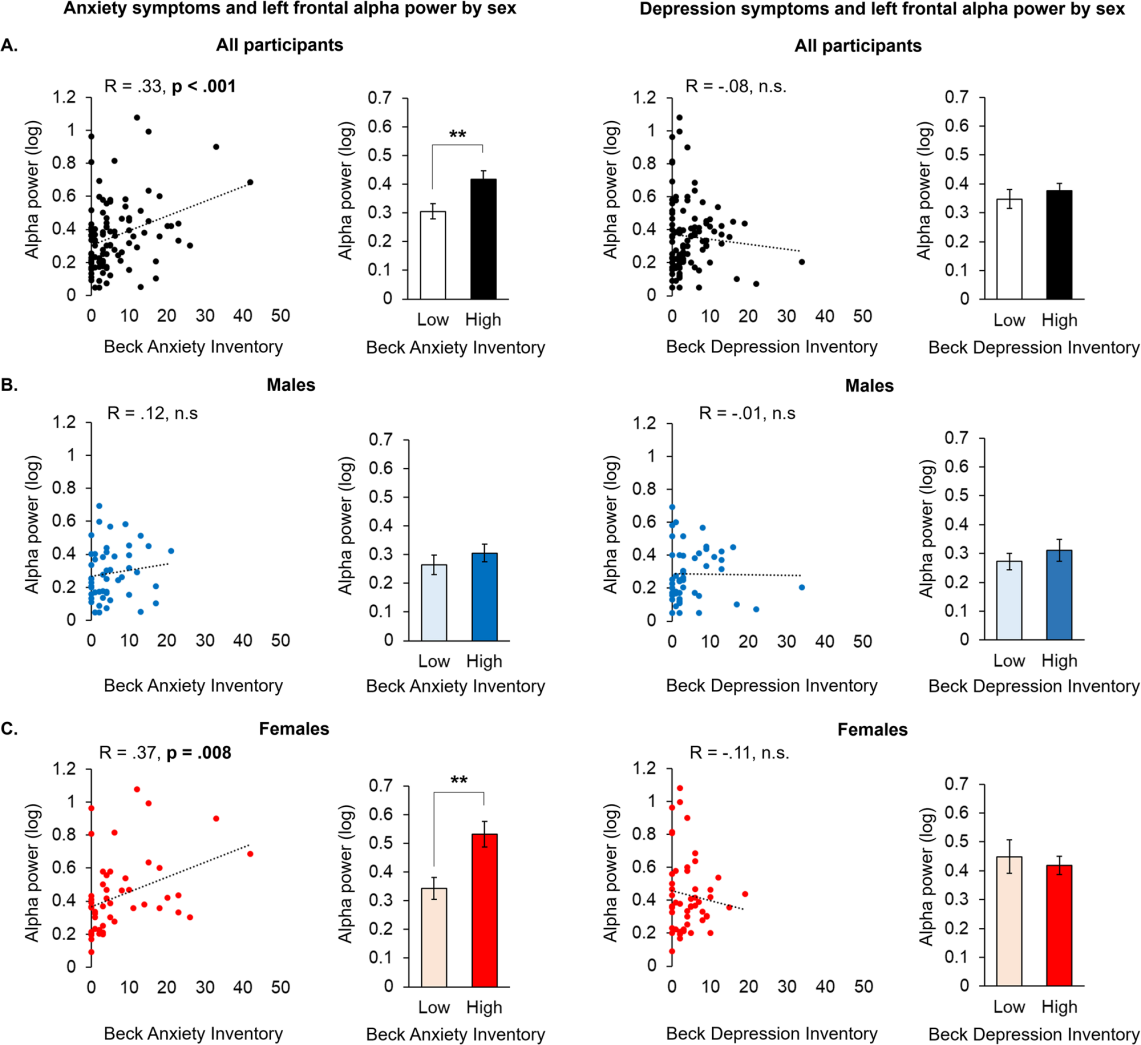
Left frontal alpha power increases as a function of past month anxiety in a sex-dependent manner. Scatter plots and median split bar graphs of data from all (**A**) male (**B**) and female (**C**) participants showing associations between alpha power under the AF7 sensor relative to scores on the Beck Anxiety Inventory and Beck Depression Inventory (*n* = 100; **p < 0.01).

In the same study, we compared associations in alpha power under the AF7 sensor to BDI and BAI scores, finding significant correlations with BAI, but not BDI (*r* = 0.33; *p* < 0.001) (Fig. 3A). A median split on BAI scores further indicated a significant difference in alpha power between individuals with “Low” (mean: 1.08; SD: 1.21) relative to “High” (mean: 11.37; SD: 8.16) BAI (*p* = 0.008; Fig. 3A). Although moderation analyses found no interaction between the broadband prefrontal variable on the relationship between BAI and alpha power under AF7 (*p* = 0.186), the relationship between BAI and alpha power under AF7 was significantly strengthened as a function of recent cannabis use (*p* = 0.037; Supplementary Fig. 4). We then examined sex- and age-related relationships with BAI scores under the AF7 sensor, finding that the association between left prefrontal alpha power and past week anxiety was present only for females (Fig. 3C), but not for males (Fig. 3B). In examining age, there was a significant, moderate relationship between BAI scores and alpha power under the AF7 electrode in females aged 24–29 (*r* = 0.61, *p* = 0.013; Supplementary Fig. 2B). Prior reports have also calculated right-minus-left prefrontal alpha power as a measure of left prefrontal activity related to anxiety, which here was less sensitive to BAI than AF7 alone (AF8 minus AF7, *r* = −0.20; *p* = 0.047) [29].

### Study 2

**Fig. 4:**
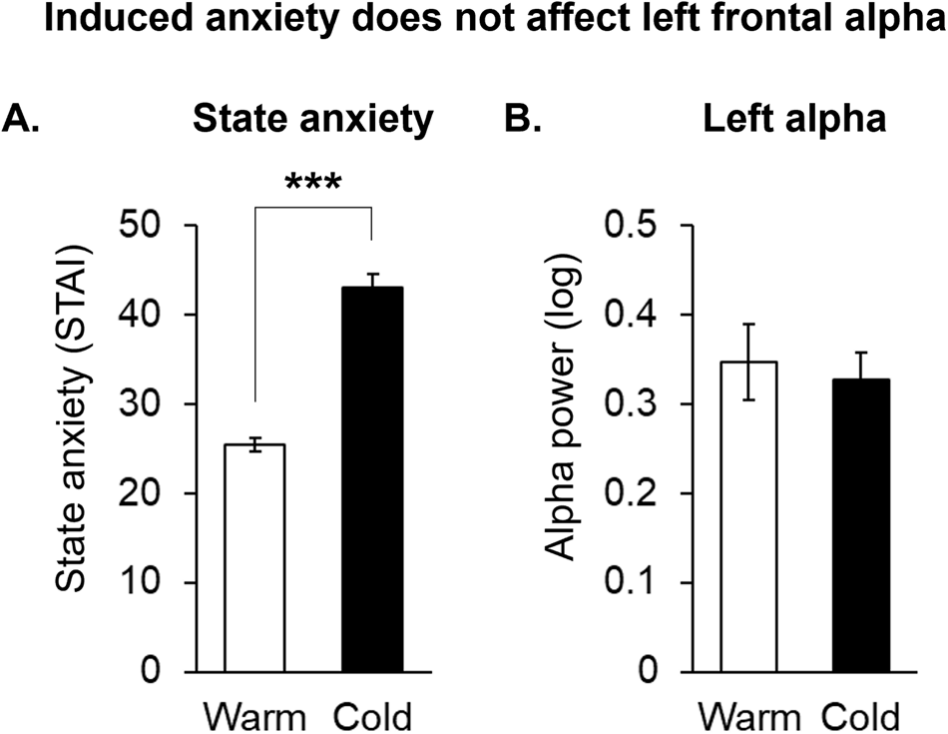
Left frontal alpha power is not modulated by state anxiety. Self-reported state anxiety from the State-Trait Anxiety Inventory (STAI) (**A**) and alpha power under the AF7 sensor (**B**) between warm and color water conditions of the cold pressor test (*n* = 40; ***p < 0.001).

Based on the above findings from the first cohort of participants linking left frontal alpha to anxiety (Study 1), we designed a second study (Study 2) with a separate cohort of participants to further probe this relationship. The demographics for these participants are shown in Table 2. Specifically, in Study 2, we explored whether cold-water immersion during the CPT would induce acute anxiety and elicit changes in AF7 alpha power. We found that the cold water condition of the CPT reliably increased state anxiety in these participants (*p* < 0.001; Fig. 4A). However, AF7 alpha power remained unchanged between the warm- (baseline) and cold-water conditions (Fig. 4B). Furthermore, recent cannabis use did not moderate the relationship between AF7 alpha power and state anxiety (*p* > 0.05).

**Table 2:** Demographic characteristics of participants in Study 2, which used mobile EEG during the warm- and cold-water conditions of the cold pressor test (CPT).

Category	*N* or Mean ± SD (Range)
Subjects (Male/Female)	50 (24/16)
Age, Years	30 ± 8 (21–48)
Body Mass Index, kg/m^2^	25 ± 4 (18–38)
Cannabis use	
Age first use, Years	18 ± 6 (11–46)
Years of use, Years	12 ± 8 (1–29)
Total lifetime use, Days	1850 ± 2034 (10–10,585)
Average days of use per year, Days	187 ± 99 (12–360)
CUDIT-R score	11 ± 5 (1–24)
Alcohol use (% weekly)	80%
MAST score (of weekly)	1.5 ± 2 (0–7)
Drinks per week (of weekly)	5.6 ± 5 (0–20)
Tobacco use (% daily)	12.5%
Cigarettes/day (of daily)	2.8 ± 4 (1–10)
Psychiatric self-assessment	
Beck Depression Inventory	3.6 ± 3.1 (0–12)
Beck Anxiety Inventory	3.4 ± 4.3 (0–19)
Trauma score	1.3 ± 1.3 (0–6)
Yale psychosis score	18 ± 6.9 (12–34)

*CUDIT-R* cannabis use disorder identification test – Revised, *MAST* michigan alcohol screening test.

## Discussion

We sought to determine whether markers of cognitive and emotional health in mobile EEG relate to measures of cannabis use and mood. We also sought to determine whether these relationships occurred in a sex- and age-dependent manner. Our main finding was that cannabis use was associated with prefrontal broadband power, as hypothesized, but more specifically, we found this association was strongest for the right prefrontal cortex and driven by young males. In addition, we found that left prefrontal alpha power was specifically related to anxiety, and not depression, and was driven by female participants. Further, we did not detect modulations of left prefrontal alpha power during the CPT, suggesting that these putative biomarkers of mental health may be more closely associated with individual differences in affective processing rather than transient task-evoked changes.

Characterizing biomarkers of mental health in healthy individuals that use cannabis is timely given significant and global increases in cannabis use in recent years [32]. Our identification of sex- and age-dependent effects of cannabis on brain markers, despite similar rates of cannabis use across the sex and age groups, underscores the role that individual variability plays between cannabis use and brain health. The youngest group we examined, ages 21–23, is near to the period of adolescence, a known risk factor for cannabis-related harms to brain health [4, 33–35]. Following repeated cannabis use, adolescents show greater impairments in executive functioning than adults [36], while age of initiation of cannabis use has been linked to smaller hippocampal volumes [37]. During adolescence, widespread structural and molecular changes in the brain include the expression of the endocannabinoid system, which governs and shapes circuits critical to cognitive and emotional health [38–40]. During this time, cannabinoid receptors reach peak expression levels prior to declining in adulthood [38, 39]. Preclinical studies have shown that repeated cannabinoid exposures during adolescence impairs the maturation of the prefrontal cortex in rats [17]. In humans, a recent review concluded that studies examining repeated cannabis use resulted in no consistent pattern of effects on resting state EEG [41]. Here, we specifically leveraged a putative biomarker related to neurodevelopment [6] to aid in translating the preclinical findings [17] to individuals that use cannabis.

Prior research has also shown that females are sensitive to the brain and behavioral health effects of anxiety [42–44]. A recent study using magnetic resonance spectroscopy found that for females between the ages of 10 and 25, anxiety was associated with reduced activity in the left prefrontal cortex, via increases in gamma-aminobutyric acid (GABA) signaling [45]. In males, GABA levels were not related to either self-reported or physiological measures of anxiety [46]. In EEG studies, individuals with altered mood have shown reduced activity in the left prefrontal cortex relative to controls [8]. For example, females with greater left prefrontal activity showed more positive affect alongside less negative affect, highlighting the role of the left prefrontal cortex in maintaining positive mood [47], although increases in right prefrontal activation have also been associated with reduced anxiety in females [48]. The current work helped to further refine markers of emotional health by showing that ratings of anxiety, but not depression, are associated with reduced activity in left prefrontal cortex measured by alpha EEG, and that females drove this association. Future studies in people without cannabis use history are needed to determine the generalizability of this finding.

We also tested whether inducing a state of anxiety would induce the same effect on the brain that we had detected in relation to the BAI. Our induction of state anxiety did not affect alpha power in the left prefrontal cortex. In support of this finding, a review of EEG studies that compared the induction of mood states relative to mood conditions found that negative moods induced by tasks did not result in the same patterns of brain activity as negative mood conditions [8].

The current work includes several important limitations. While our study sought to examine the utility of mobile EEG devices, our research was limited by the capabilities of the mobile devices, which only include two prefrontal sensors and two lateral sensors. However, concerns related to the quality of EEG data obtained from mobile EEG studies may be offset by the quantity of data obtained through the ease of mobile procedures across a large number of participants. Measures of cannabis use relevant to brain health are limited by the timeframe examined and potential for introducing confounds, including age-related confounds in measures of years of cannabis use. Our operationalization of cannabis use, as average yearly cannabis use, was specifically designed to capture chronic cannabis use without introducing age-related confounds, toward maximizing relationships between cannabis use and EEG measures. Our study was also limited by the specific population we examined, individuals that use cannabis. Future studies should examine individuals that use other substances, such as alcohol or cocaine, to shed light on whether frontal broadband EEG is specific to cannabis use, generalizable to substance use broadly, or even represents the severity of substance dependence.

Together, our work helped to validate and refine putative biomarkers of cognitive and emotional health in individuals that use cannabis. We report that cannabis use was inversely related to a marker linked to neurocognitive development, aiding in the translation of preclinical findings of impaired prefrontal maturation following repeated cannabinoid exposures. In addition, we found that left prefrontal alpha power was related to past week anxiety, but not anxiety induced experimentally. Our findings occurred in a sex- and age-related manner, with young males showing greatest sensitivity to cannabis, and females showing greatest sensitivity to anxiety. The findings advance the utility of EEG to examine biomarkers of brain and mental health both inside and outside of laboratory settings.

### Citation diversity statement

The authors have attested that they made efforts to be mindful of diversity in selecting the citations used in this article.

## Supplementary information

SUPPLEMENTAL MATERIAL

## Data Availability

Raw datasets used in the analyses are available from the corresponding author upon request.
