# Supplementary material for: Mobile EEG examination of putative biomarkers of mental health in individuals that use cannabis
Source: NPP Digit Psychiatry Neurosci. 2025 Jul 3;3:18. doi: 10.1038/s44277-025-00039-8 (PMC12226337; doi:10.1038/s44277-025-00039-8)
Supplement: Supplementary file 1 — SUPPLEMENTAL MATERIAL [file 44277_2025_39_MOESM1_ESM.docx]

**SUPPLEMENTAL MATERIAL**

**
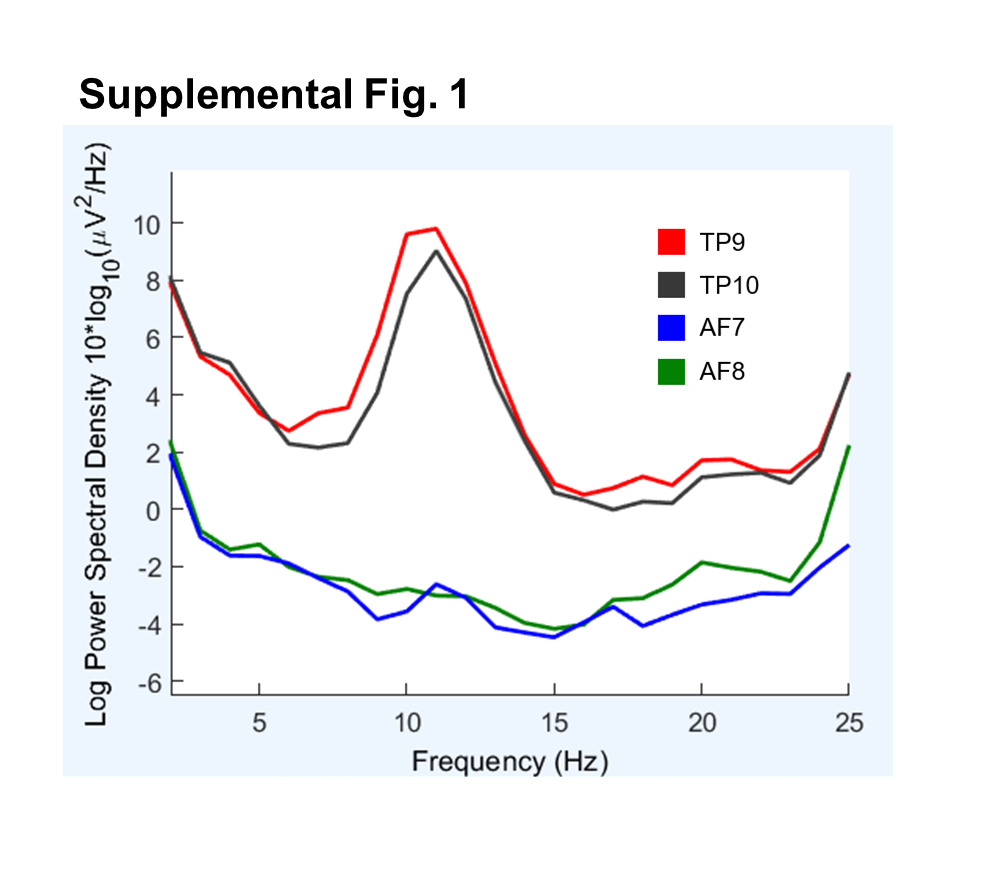
**

**Supplemental Figure 1.** Spectral power density of the four sensors of the Muse S headband in a single, volunteer during 5 minutes of eyes closed rest. Anterior prefrontal (AF) 7 and AF8 sensors record activity under the left and right prefrontal cortex, respectively. Temporoparietal (TP) 9 and TP10 sensors record activity under the left and right temporoparietal cortex, respectively. The TP9 and TP10 sensors detected high alpha power (8 – 13 Hz).

**
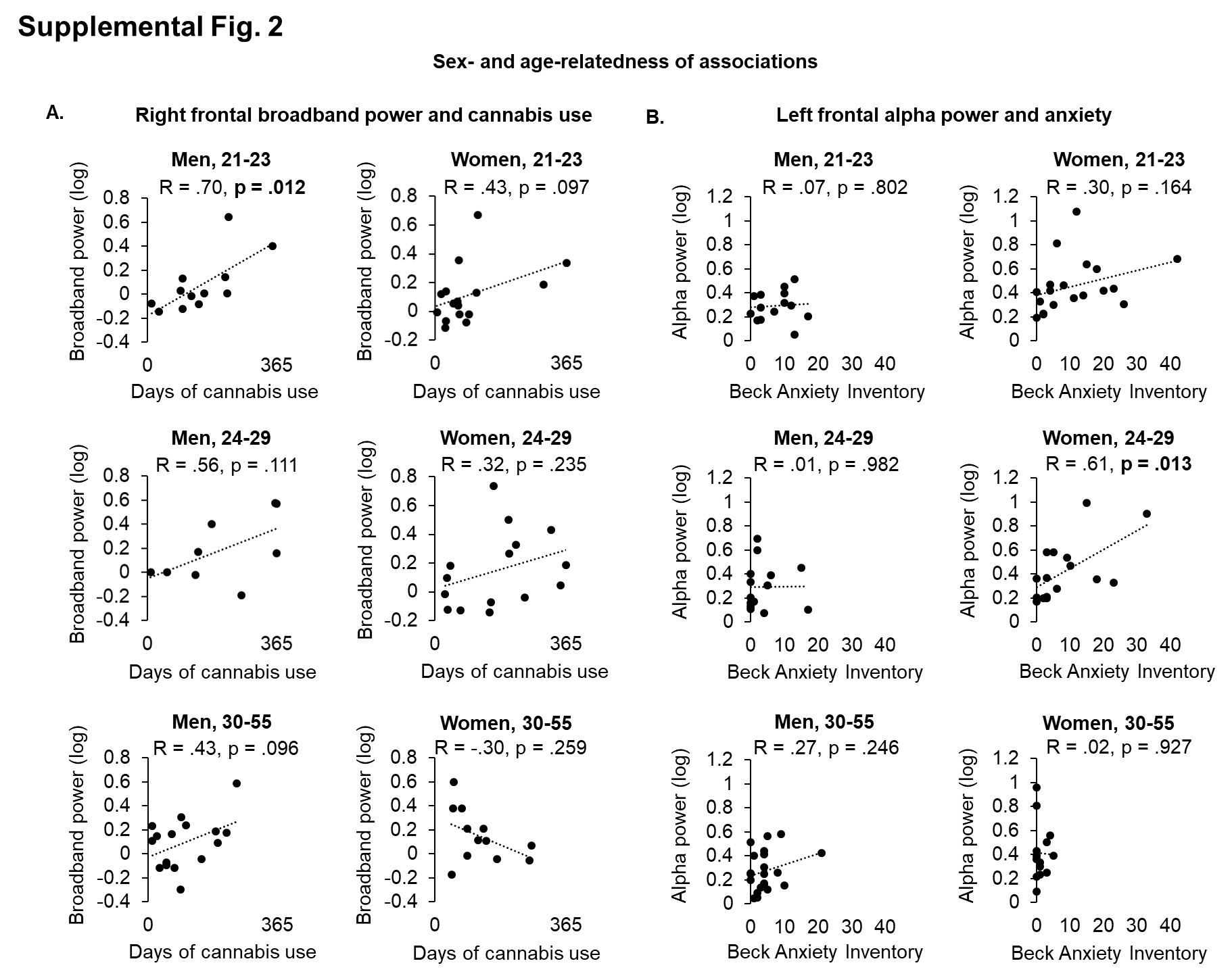
**

**Supplemental Figure 2.** Scatter plots of data from male (50) and female (50) participants across three age groups of near equivalent numbers of participants per age group. The two columns on the left show associations between broadband power under the AF8 sensor and cannabis use (A) and the two columns on the right show associations between alpha power under the AF7 sensor relative to scores on the Beck Anxiety Inventory (B).

**
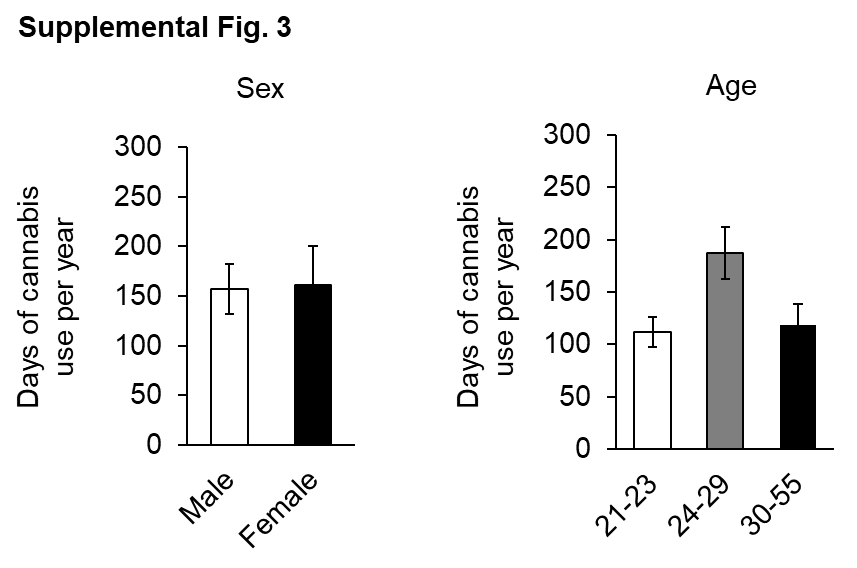
**

**Supplemental Figure 3.** Participants’ average days of cannabis use per year since onset did not differ between males and females in a t-test (A) or between the youngest (ages 21-23) and oldest (ages 30-55) individuals in a t-test (B), p > 0.05.

**
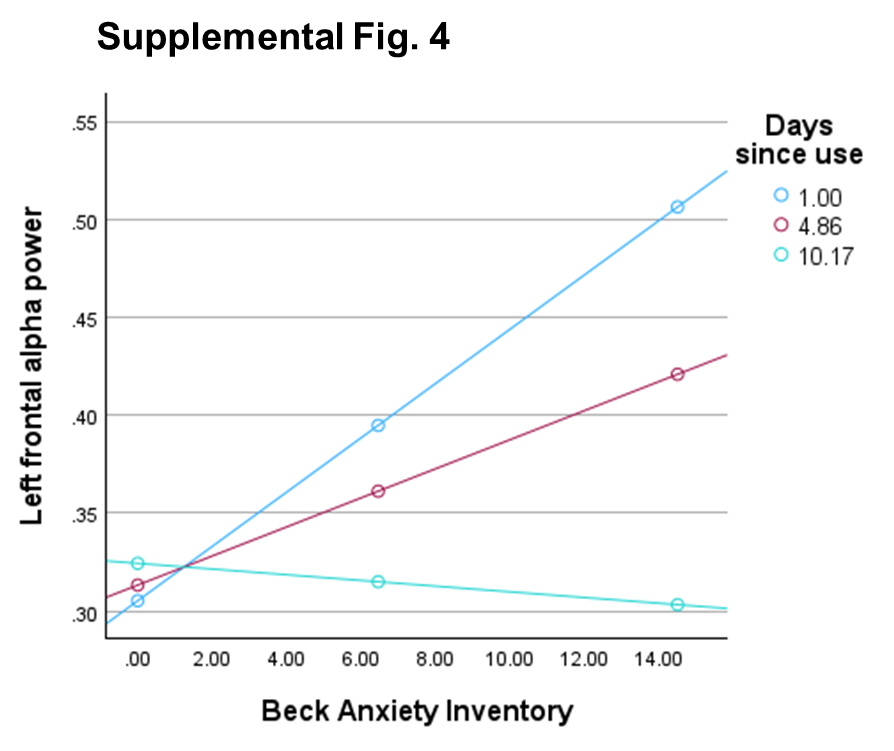
**

**Supplemental Figure 4.** Moderation analysis. There is a significant interaction between Beck Anxiety Inventory and recency of cannabis use (Days since use) (β = -0.002, SE = 0.001, t = -2.12, p = 0.037), indicating that the relationship between Beck Anxiety Inventory and left frontal alpha power was moderated by recent cannabis use. The simple slope of Beck Anxiety Inventory on the recency of cannabis use (Days since use) was significant at low (-1 SD) days since use, i.e., 1.00 days, (β = 0.14, SE = 0.001, t = 4.14, p < 0.001) but not at a longer (+1 SD) days since use, i.e., 10.17 days, (β = -0.001, SE = 0.001, t = -0.26, p = 0.795), indicating that individuals who have used cannabis more recently show a stronger relationship between Beck Anxiety Inventory and left frontal alpha power.

**
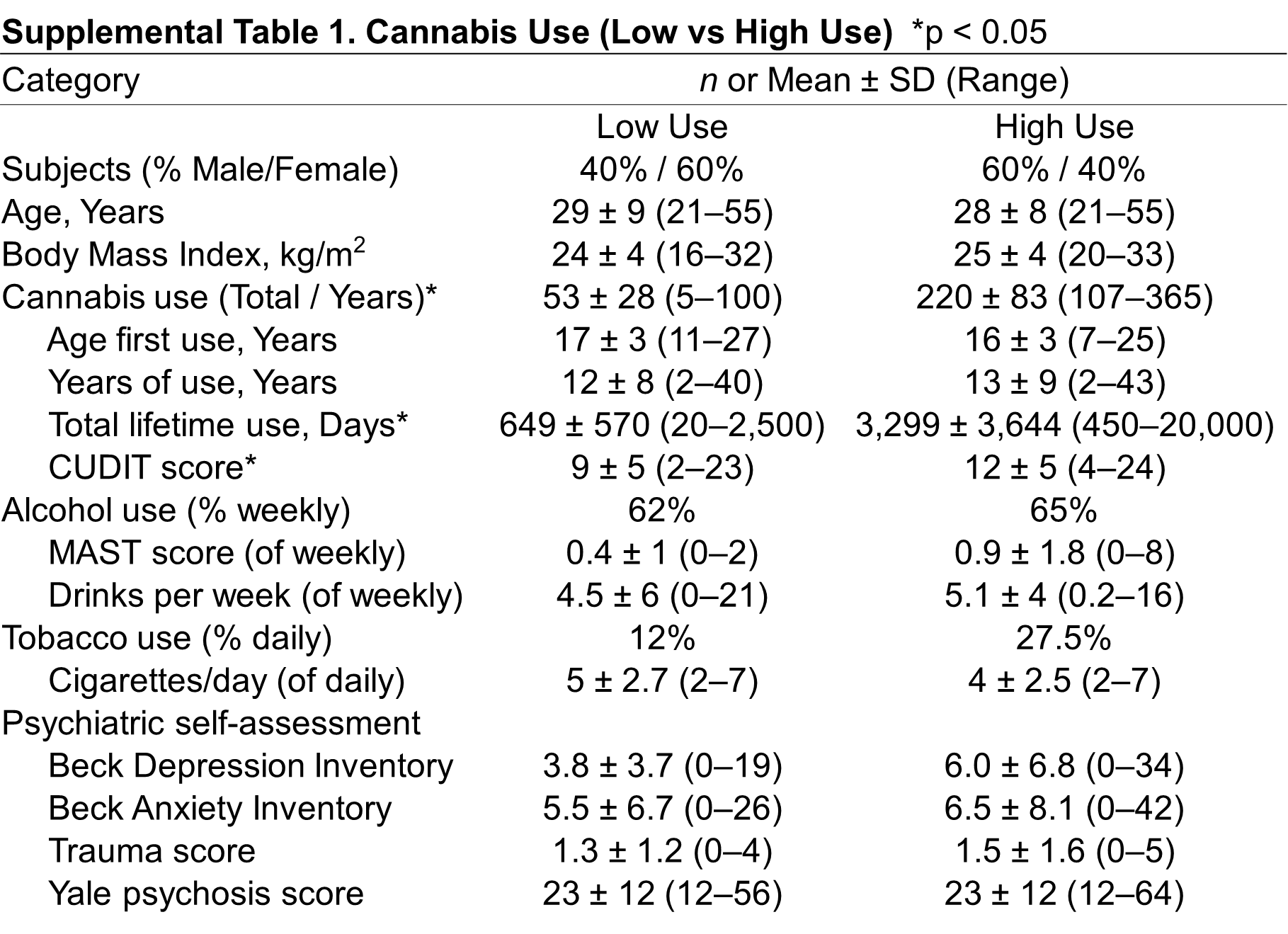


Supplemental Table 1**. Demographics of the Low and High cannabis use groups. CUDIT = Cannabis Use Disorder Identification Test; MAST = Michigan Alcohol Screening Test, short form.
